# What Has SARS-CoV-2 Taught Us About Evolution?

**DOI:** 10.7759/cureus.94502

**Published:** 2025-10-13

**Authors:** Yingguang Liu

**Affiliations:** 1 Molecular and Cellular Sciences, Liberty University College of Osteopathic Medicine, Lynchburg, USA

**Keywords:** adaptation, adaptive radiation, covid-19, diminishing returns, epistasis, muller’s ratchet, origin and evolution of viruses and other micro-organisms, pleotropic effects, punctuated equilibrium theory, sars-cov-2 (severe acute respiratory syndrome coronavirus-2)

## Abstract

Over the past five and a half years, SARS-CoV-2 has demonstrated in real time many concepts and principles of evolutionary biology. Soon after it was disseminated globally, the virus underwent adaptive radiation, resulting in the generation of multiple dominant variants. Later variants drove earlier ones to extinction in a series of selective sweeps. The nature of adaptation was shifting molecular specialization, with the spike protein losing binding affinity toward bat cells to gain affinity toward human cells, losing replicative fitness in lung cells to gain fitness in nasal cells. Evolution of the spike protein was constrained between two beneficial results: enhancing receptor binding and evading neutralizing antibodies. Because there are limited ways of functional improvement, multiple variants converged on the same spike mutations, with higher-impact mutations fixed before lower-impact mutations, giving a new meaning to diminishing-returns epistasis. Later genetic changes became repetitive and cyclical. The Delta variant represented an evolutionary dead end. Evolution of the virus also demonstrated punctuated equilibrium, with saltatory changes producing highly mutated variants, which subsequently experienced gradual structural and functional drifts. While structural proteins experienced strong positive and purifying selections, nonstructural and accessory proteins accumulated neutral and deleterious mutations, most of which remain unfixed. Selection of adaptive missense mutations resulted in deoptimization of codon usage. These phenomena point to Muller’s ratchet in action. The higher codon usage score in the initial Omicron variant was probably due to long-term preservation of the virus in an immunocompromised host, where low immune pressure prevented genetic degradation.

## Introduction and background

With their high mutation rates, short generation times, large effective populations, and strong selection forces, viruses provide effective models to study evolutionary processes, especially in the genomic era [1]. SARS-CoV-2, the causative agent of COVID-19, far surpasses every other organism in terms of the number of individual genomes studied. Over 17 million genomic sequences have been deposited in the GISAID database in 5.5 years of the pandemic [2]. With the fitness effect of its genetic mutations intensively studied and its interaction with the human host closely monitored, the evolutionary trajectory of SARS-CoV-2 has been unfolding before the biological community. We have observed its rapid diversification, succession of variants, selective sweeps, and “extinction” of once dominant lineages [3]. Consequently, the clinical manifestations of COVID-19 also evolved at a pace that has not been seen in other diseases [4-7]. Interestingly, there are signs that the evolution of SARS-CoV-2 is slowing down [8,9]. The purpose of this paper is to review the evolutionary patterns of SARS-CoV-2 and outline the lessons that the virus has taught us about biological evolution in general.

## Review

Rapid adaptive evolution in a new host species

Animal viruses tend to live commensally in their natural hosts. For example, an average human being harbors eight to 12 viruses in his/her body [10]. However, they often cause diseases if transmitted to another species. Examples of commensal animal viruses that cause severe diseases in humans include the monkey B virus and the simian/human immunodeficiency virus (HIV) [11,12]. Likewise, coronaviruses usually cause no symptoms in their reservoir species, bats, but are pathogenic in humans [13,14]. When a virus encounters a new host species, it must adapt to the new cellular machinery and fight the new immune system to survive. Some viruses fail to adapt and are expelled by the new host species. Examples include avian influenza viruses, which are not transmitted between people even though humans are occasionally infected [15]. SARS-CoV-1 and Middle East respiratory syndrome coronavirus (MERS-CoV) can transmit between people, but so far have failed to establish human reservoirs [16]. Other zoonotic viruses, such as SARS-CoV-2 and HIV, have taken root and thrived in human populations.

Viruses can afford error-prone replication enzymes to explore potentially beneficial genetic changes. In the past 5.5 years, SARS-CoV-2 has undergone an amount of evolution that is comparable to that of the human species since it diverged from chimpanzees (Table 1). Although there is evidence of fast evolution of other zoonotic viruses such as the H1N1 influenza A viruses, SARS-CoV-1, and HIV-1 after they entered mankind, we have not seen viral evolution at such a large scale or with such real-time documentation [17,18].

**Table 1:** Comparison between the evolutionary histories of SARS-CoV-2 and human *Genome size and mutation fitness effect are not considered here because they tend to cancel each other. Viruses have smaller genomes and accumulate fewer mutations, but each mutation affords stronger fitness effects.

Parameter	SARS-CoV-2	Human*
Mutation rate (per nucleotide per generation)	1-2x10^-6 ^[19]	1-2x10^-8 ^[20]
Generation time	8-24 hours [21]	26.9 years [22]
History	5.5 years	7 million years
Generations	2,000-6,000	260,000
Total mutations per nucleotide	2x10^-3^-1.2x10^-2^	2.6 10^-3^-5.2x10^-3^

Adaptive radiation and selective sweeps 

A new zoonotic virus entering humanity can be compared to a new species arriving on a previously uninhabited island. Successful adaptation often leads to rapid diversification into multiple new forms. In the case of SARS-CoV-2, diversification was not driven by selective environments of different niches but by an unfavorable environment of the same niche, suboptimal cellular machinery, and a hostile immune system. The only advantages the virus had were airborne transmission and ambulatory hosts. Adaptive radiation manifested as rapid fixation of new mutations leading to the emergence of over a dozen dominant variants (including Alpha to Mu, designated by the World Health Organization) within the first 13 months of the pandemic (from December 2019 to January 2021). Subsequently, competition for the same niche resulted in a series of selective sweeps, driving most new forms to extinction. Within the first six months of the pandemic, the D614G mutant had replaced the original virus in the first selective sweep [23]. Noticeably, all Variants of Concern (VOCs) and the early Variants of Interest (VOIs) had unique mutation spectra, which derived directly from the original virus; none of them evolved from a previous VOC or VOI. By early 2022, the Omicron VOC had replaced all other variants to become the sole remaining form of SARS-CoV-2.

**Figure 1:**
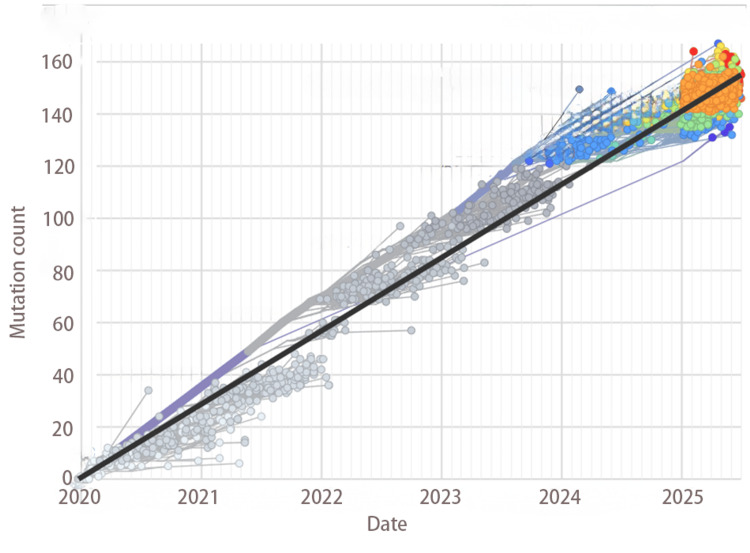
Mutation fixation in SARS-CoV-2 clades over time (nextstrain.org)

The Omicron variant continued to evolve, leading to a succession of dominant subvariants. However, mutations became increasingly cyclic and repetitive [24]. Since late 2023, the evolution of SARS-CoV-2 seems to have slowed down as the accumulation of fixed mutations plateaued (Figure 1) [8,9]. There has been no new VOC or VOI since the emergence of Omicron JN.1 in August of 2023. If selective sweeps do occur in the future, we expect more soft sweeps (fixation of preexisting mutations) rather than hard ones (fixation of a single new mutation with a large fitness effect). See glossary in Appendices for more explanation of hard and soft selective sweeps.

The molecular nature of adaptation

On the molecular level, adaptation often involves some change in the specificity of protein interactions, such as receptor-ligand binding, enzyme-substrate binding, and antibody-antigen binding. Some of the binding proteins are built with inherent genetic or epigenetic plasticity, such as the DNA recombination and somatic hypermutation mechanisms in antibody production, the population polymorphism of glutathione S-transferase (GST) enzymes, and the epigenetic induction of drug-metabolizing enzymes [25-27]. In mutational adaptation of enzymes, a gain in binding affinity toward a new substrate is often accompanied by a loss of binding affinity toward the original substrate [28]. 

The most notable aspects of adaptive evolution of SARS-CoV-2 are receptor binding and antibody evasion. The former involves optimization of binding between the viral spike protein and its receptor on the host cell membrane, angiotensin-converting enzyme 2 (ACE2). Early in the pandemic, it was found that SARS-CoV-2 demonstrated lower binding affinity toward bat ACE2 than the human homolog [29]. Moreover, the virus had lost the ability to replicate in bat cells unless the cells were engineered to express human ACE2, indicating rapid adaptation soon after entering humanity, or the virus had pre-adapted in an intermediate environment [30]. Binding affinity toward bat ACE2 further declined in the Lambda and Omicron variants as the virus evolved [31]. Meanwhile, binding affinity toward human ACE2 increased in most VOCs. Partly because of the higher concentration of the human ACE2 in the airway epithelium than in the lungs, later variants of SARS-CoV-2 increasingly infected the upper respiratory tract (nasal epithelium) while losing replicative fitness in lung cells (via a variety of mechanisms), accounting for the decreased frequency of severe pneumonia [32-35].

Another major driving force of SARS-CoV-2 evolution, especially later in the pandemic, is evasion of neutralizing antibodies. The spike protein, especially its receptor-binding domain (RBD), is the major target of neutralizing antibodies [36]. Unlike receptor binding, where there are optimal binding modes to strive toward, immune escape mutations are not necessarily directional. A structural change is beneficial as long as it reduces binding affinity toward the dominant antibodies of the time. However, because the spike protein presents a limited number of accessible, immune-dominant epitopes and a limited number of common immunoglobulin genetic segments are involved in recognizing these epitopes, immune evasion mutations of SARS-CoV-2 are restricted to a limited set of amino acid residues in the spike protein [37-39].

Besides receptor binding and immune escape, other factors affect the replicative fitness and epidemiological fitness of SARS-CoV-2. For example, the D614G mutation resulted in an RBD that bound less tightly to ACE2 than wild type, but the mutation increased the density of intact S trimers on the viral surface by preventing premature dissociation of S1 from S2 following furin cleavage in the producing cell [40,41]. The preference of the nasal epithelium over the lungs made viral shedding easier, resulting in shorter incubation periods and more effective transmission [42].

The constraints of adaptation

Deleterious Pleiotropic Effects

In a 2024 review paper, Yao and colleagues reported 43 immune escape mutations in the spike protein, 14 of which simultaneously enhanced infectivity/transmissibility of the virus, while 18 facilitated immune escape at the cost of infectivity/transmissibility [43]. Immune escape mutations that reduced infectivity/transmissibility exemplify deleterious pleiotropic effects [44,45]. The trajectory of SARS-CoV-2 evolution seemed to oscillate between maintaining ACE2-binding and immune evasion [46,47]. 

While in vitro evolution using yeast display of RBD variant libraries yielded one best model of receptor binding (RBD-62) with a binding affinity that is 1000-fold stronger than that of wild type [48], the highest affinity the virus ever achieved in nature was no more than 20-fold stronger than wild type [8]. The strongest receptor-binding variant in various reports was BA.2.75, XBB.1.5, or BA.2.86 (Omicron subvariants identified between 2022 and 2023), depending on measurement methods and samples analyzed [35,49-52]. BA.2.86 and its descendants had five of the nine affinity-enhancing mutations in RBD-62 plus V445H/R, which is similar to V445K in RBD-62 (Table 2). The other three mutations found in RBD-62 were never fixed in natural variants, suggesting that these amino acid residues must be conserved for effective viral replication or for infection of the human host.

**Table 2:** In vitro and natural evolution of SARS-CoV-2 RBD Data were obtained from references [48] and [53]. Standard single-letter codes are used for amino acids: I for isoleucine, V for valine, G for glycine, N for asparagine, T for threonine, S for serine, E for glutamic acid, F for phenylalanine, Q for glutamine, K for lysine, R for arginine, M for methionine, Y for tyrosine, H for histidine, P for proline, and A for alanine. Greek transliterations represent SARS-CoV-2 variants designated by the World Health Organization. Pango designations are used for Omicron subvariants. RBD, receptor-binding domain

RBD position		358	445	446	448	460	468	470	477	478	481	483	484	490	493	494	498	501
WT residue		I	V	G	N	N	I	T	S	T	N	V	E	F	Q	S	Q	N
Observed in in vitro evolution products		F	K	R	S	K	T/V	M	N	S	Y	E	K	Y/S	H	P	R	Y
In vitro product Clone B62		F	K			K	T	M	N				K				R	Y
Alpha																		Y
Beta													K					Y
Gamma													K					Y
Delta										K								
Zeta													K					
Eta													K					
Theta													K					Y
Iota									(N)				(K)					
Kappa													K					
Lambda														S				
Mu													K					Y
Omicron	BA.1			S					N	K			A		R		R	Y
	BA.2								N	K			A		R		R	Y
	BA.4								N	K			A				R	Y
	BA.5								N	K			A				R	Y
	BQ.1					K			N	K			A				R	Y
	BA.2.75			S		K			N	K			A				R	Y
	XBB.1.5		P	S		K			N	K			A	S	R		R	Y
	BA2.86		H	S		K			N	K	K		K				R	Y
	JN.1		H	S		K			N	K	K		K				R	Y
	KP3.1		H	S		K			N	K	K		K		E		R	Y
	LP.8.1		R	S		K			N	K	K		K		E		R	Y

The Order of Mutation Fixation and Diminishing Returns

When a physiological function is optimized via adaptive genetic changes, beneficial changes follow a trend of diminishing returns. Experimental studies showed that the same beneficial change yielded less fitness effect when it occurred after the genetic background had been improved by other changes [54-56]. In other words, earlier changes exert a negative epistatic effect over later changes.

The evolution of SARS-CoV-2 demonstrated another trend of consecutive beneficial changes: mutations with larger fitness effects are fixed before mutations with smaller fitness effects. Because the ways to improve a function are limited, the fitness effect of a mutation declines over time. Table 2 shows that multiple SAR-CoV-2 variants independently converged on a mutation spectrum that was predicted by the best in vitro evolution product, IBD-62 [48]. N501Y strongly enhanced receptor-binding affinity while E484K moderately enhanced receptor-binding but strongly reduced antibody binding [57,58]. Both mutations were fixed independently in multiple variants early in the pandemic. Omicron initially had E484A but switched to K484 in BA.2.86 [59]. Q498R reduced ACE2-binding affinity by itself, but significantly enhanced binding in the presence of N501Y (positive epistasis) [60]. A combination of Q498R and N501Y appeared in multiple in vitro evolution products but was fixed in nature only in Omicron after two years into the pandemic [48]. S477N moderately stabilized the RBD-ACE2 interaction and was detected in earlier variants (e.g., Iota) but fixed in Omicron only [48,61]. N460K was found to enhance ACE2 binding in digital simulations but not experimentally [62-64], and its appearance and fixation in multiple Omicron subvariants in 2022-2023 were more likely due to its immune escape effect [64,65]. T478K enhanced ACE2-binding modestly and facilitated immune escape [66,67]. It was not predicted by in vitro evolution but was fixed in the Delta and Omicron variants. Although the emergence of most of the RBD mutations was largely path-independent, the order of their fixation followed a general trend from high-impact mutations to low-impact mutations.

With diminishing fitness gains, mutation accumulation in SARS-CoV-2 slowed down as replicative fitness approached optimum. Within-clade amino acid substitution rate quickly dropped from 10-15/year to 3-9/year [68]. The overall interclade substitution rate also showed a trend of decline, especially after the advent of JN.1 in 2023 (Figure 1). 

*Path Exclusion* 

The Delta VOC followed a different path, which led to a “dead end.” It contained only two mutations in the RBD, L452R and T478K. The former strongly enhanced ACE2-binding and synergized with the latter [69,70]. It did so partly by pushing Q493 closer to the N-terminal helix of ACE2 to form hydrogen bonds with multiple ACE2 residues. This understandably constrained the mutability of Q493 (Q493H enhanced ACE2-binding in vitro while Q493R was fixed in multiple Omicron subvariants) [70].

There appeared to be a mutual negative epistasis between L452R and N501Y [60]. In the Delta background, the effect of N501Y on ACE2 binding is reduced. In the presence of N501Y (all other VOCs), L452R dampened ACE2-binding instead of enhancing it (sign epistasis).

Delta outcompeted earlier variants because of its unique mutations that enhanced receptor binding and facilitated immune escape, but when Omicron came on stage, Delta failed to adapt further and was driven to extinction, while Omicron evolved toward the optimum configuration of RBD-62. The BA.4 and BA.5 subvariants of Omicron re-acquired L452R along with compensating mutations, including the R493Q reversion, but they were soon outcompeted by other Omicron subvariants (such as XBB.1.5) that did not have L452R.

In addition, L452R is mutually exclusive with F490S. The latter was predicted by in vitro evolution and fixed in the XBB subvariants [48,71].

“Yoyo Mutations”: Recycling the Same Tricks

L452R and Q493R represent two of the seven “yoyo mutations” of SARS-CoV-2, which appeared and disappeared multiple times on the mutant landscape [24]. As mentioned above, L452R was anti-correlated with Q493R. The latter appeared in Omicron subvariants BA.1 and BA.2 but reversed in BA.4 and BA.5 when they re-acquired L452R. Q493R enhanced ACE2-binding in the absence of L452R, but the reversal increased receptor-binding affinity in the presence of L452R (another case of sign epistasis) [72]. The combination of R493Q and L452R allowed BA.4 and BA.5 to gain immune escape ability without sacrificing receptor binding [73].

Q493H was predicted by in vitro evolution, and it was partially fulfilled by Q493R since R and H are both basic. However, the mutation of Q493 into glutamic acid (E) in recent Omicron subvariants (e.g., KP.3 and LP.8) came as a surprise, not only because it was electrostatically contrary to Q493H and Q493R, but because most other mutations in the RBD resulted in basic amino acids K, R, or H. However, Q493E increased ACE2-binding affinity in the presence of L455S and F456L in KP.3 and LP.8, as it formed local salt bridges with ACE2 H34 and K31 while avoiding electrostatic repulsion from ACE2 E35 [74,75].

Evidently, some of the temporal “reversions” were not true reverse mutations but were due to alternations of dominant variants. For example, the Δ69-70 mutation was present in Alpha, absent in Delta, present in BA.1, and absent in BA.2, but Delta and BA.2 did not regain the two deleted amino acids. They never lost them. Most current Omicron subvariants (JN.1 descendants and a few recombinants) have Δ69-70, indicating H69V70 has probably been deleted permanently from the spike protein.

The “yoyo” mutations created moving targets, allowing the virus to dodge host immunity. As the virus gained sufficient affinity toward the human ACE2, it could afford to mutate antibody targets back and forth depending on the dominant antibody profile of the time. The FLip mutations (L455F+F456L) in recent Omicron subvariants apparently served the same purpose of immune evasion while preserving receptor-binding affinity [76].

Punctuated “Equilibrium”

The evolution of SARS-CoV-2 was marked by three periods of saltatory changes followed by gradual accumulation of mutations. The first period was the adaptive radiation between July 2020 and January 2021, when the virus diversified rapidly, giving rise to all the VOCs and VOIs from Alpha to Mu. Although we saw no more than three fixed mutations in the RBD in each of these variants, fixed mutations throughout the spike protein and the entire genome were numerous and diverse. For example, Gamma fixed 12 mutations in the spike while Delta fixed 29 mutations throughout the genome. The second evolutionary burst was the appearance of Omicron in December 2021, with its 30+ mutations in the spike and 50+ in the entire genome. The third evolutionary burst happened in BA.2.86, which was identified in August 2023 when the total number of mutations in the spike protein jumped to 57 and the total genome mutations jumped to over 90 [53]. The period of gradual change between the first and second bursts was 11 months, and the period between the second and third bursts was 17 months [77]. Two years have passed since BA.2.86, and there have been no more mutational bursts.

Because most of the early variants were short-lived, gradual changes were mainly observed in Omicron. The overall trend of genetic changes was the sudden addition of receptor-binding mutations followed by the gradual appearance of immune escape mutations. The main affinity-enhancing mutations in BA.1 were S477N, Q493R, Q498R, and N501Y. Synergism between Q498R and N501Y reconfigured the RBD-ACE2 interaction and allowed for the affinity-reducing immune escape mutations to accompany or follow the affinity-enhancing mutations [35,78]. BA2.86, even with its numerous affinity-enhancing mutations, did not gain dominance over the reigning XBB lineages. One more mutation, L445S, which offered immune escape at the expense of receptor-binding, turned BA.2.86 to JN.1, which quickly outcompeted all other lineages to dominate the variant landscape [51]. 

Although adaptive substitution rates have slowed down in the spike protein and throughout the genome, reversions are much fewer in number, so we have not seen an equilibrium concerning genetic changes. Functionally, however, the current JN.1 sublineage has been able to fine-tune the RBD-ACE2 interactions to maintain effective receptor-binding (with newer mutations such as R346T and Q493E) while constantly eluding herd immunity [75,79]. At this point, the evolutionary relationship between the virus and the host conforms to the Red Queen Hypothesis of relative equilibrium, setting the stage for their long-term coexistence [80].

Muller’s ratchet

Muller’s ratchet refers to the irreversible accumulation of deleterious mutations in asexual organisms as a result of genetic drift [81]. It has been experimentally verified using RNA viruses, protozoa, and *Salmonella typhimurium* through artificial bottlenecks [82,83]. Moreover, genetic degeneration has also been documented in the HIV and the influenza virus in nature, even with their large population sizes [84,85]. SARS-CoV-2 has presented us with an opportunity to thoroughly observe the effect of Muller’s ratchet in real time.

During the COVID-19 pandemic, there have been repeated instances of recombination between variants [86]. The XBB subvariants that dominated the variant landscape for over a year between 2022 and 2024 were recombinants between two BA.2 sublineages. Nevertheless, recombination is relatively rare and does not seem to affect the overall evolutionary trajectory of SARS-CoV-2.

Accumulation of Mutations in Nonstructural and Accessory Genes: Weak Selection, Drift, and Hitchhiking 

Although positive selection has been the driving force for adaptive evolution of the spike protein, purifying selection has been the stronger force on nonsynonymous mutations overall, and the fitness effect of SARS-CoV-2 mutations has become more negative over time, consistent with a gradual slowdown of adaptive evolution [8,87]. However, purifying selection only eliminated a fraction of deleterious mutations. By February of 2022 (two years into the pandemic), ~62% of the viral genome had mutated at least once, 61.5% of mutations being nonsynonymous [88]. The ratio between nonsynonymous mutations and synonymous mutations (dN/dS) is higher among the accessory genes, but is generally close to one, indicating neutral evolution of these genes [87,89-91]. Nonsense mutations are almost exclusively found in accessory genes [92].

**Figure 2:**
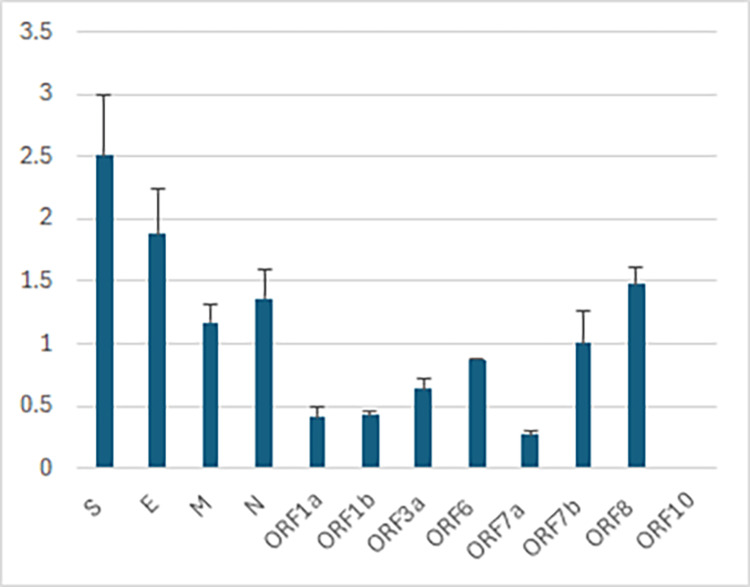
Percentage of fixed mutations among SARS-CoV-2 genes Data were obtained from references [88] and [92]. Single-letter codes are used to represent structural proteins with S for spike, E for envelope, M for membrane, and N for nucleocapsid.

I calculated the mutation fixation rates in SARS-CoV-2 genes using the total mutation counts from two reports [88,92] and the number of fixed mutations found in all variants within the same period (Table 3 and Figure 2). The results show higher fixation rates in the structural genes (S, E, M, and N) and lower fixation rates in nonstructural genes (encoded by ORF1a and ORF1b), with accessory genes (ORF3, ORF6, and ORF7-10) in between, in partial agreement with an early report [93]. Even though the nonstructural proteins are under strong purifying selection (dN/dS≈0.5) and fixed less than 0.5% of missense mutations, they still bear ~65% of the mutation load of the genome, most of which presumably are near neutral. From the strong purifying selection on the spike (S) and membrane (M) glycoproteins [91], we can assume the high fixation rates in S and M are due to adaptive evolution. Since the envelope glycoprotein (E) and the nucleocapsid (N) are under weaker purifying selection [91], we are less confident whether the fixed mutations were due to selection or drift. However, we know that the Omicron-specific E:T9I mutation renders the virus resistant to lysosomal degradation and enhances transmission fitness, so it can be reasonably attributed to positive selection [94].

**Table 3:** Total mutations and fixed mutations in SARS-CoV-2 genes Data were obtained from references [88] and [92]. Fixed mutation counts were obtained from [53] according to the latest variant of the time.

Protein	Gene size	Number of mutations according to [88]	Number of mutations according to [92]	Fixed by Feb 2022 [88]	Fixed by Oct 2022 [92]	Fixation percent according to [88]	Fixation percent according to [92]	Mean Fixation Rate	SD
Spike	3821	3799	2974	82	85	2.2	2.9	2.5	0.5
Envelope	227	185	140	3	3	1.6	2.1	1.9	0.4
Membrane	668	568	470	6	6	1.1	1.3	1.2	0.2
Nucleocapsid	1259	1512	1243	18	19	1.2	1.5	1.4	0.2
ORF1a	13176	12218	9358	42	44	0.3	0.5	0.4	0.1
ORF1b	8111	4489	4713	18	21	0.4	0.4	0.4	0
ORF3a	827	1225	1002	7	7	0.6	0.7	0.6	0.1
ORF6	185	230	230	2	2	0.9	0.9	0.9	0
ORF7a	365	823	678	2	2	0.2	0.3	0.3	0
ORF7b	131	242	169	2	2	0.8	1.2	1	0.3
ORF8	365	584	507	8	8	1.4	1.6	1.5	0.1
ORF10	116	130	121	0	0	0	0	0	0

On the other hand, mutations in ORF3a, S171L in Beta, and T223I in Omicron, impede viral replication yet were fixed in the corresponding lineages, presumably due to hitchhiking or drift [95,96]. The D61L mutation in ORF6 is also detrimental to the virus. ORF6 antagonizes antiviral interferons by disrupting nucleocytoplasmic trafficking. D61L abolishes this function and reduces replicative fitness. Nevertheless, the mutation was fixed in BA.2, BA.4, XBB, and their descendants, including the currently dominant lineages. The mutation might be responsible for the underperformance of BA.4 against BA.5, which did not have the mutation. However, the return and fixation of D61L in XBB likely hitchhiked on the epidemiological fitness of XBB over BA.5 and its sublineages, underlining the effect of linkage in the fixation of detrimental mutations.

Accessory proteins, by definition, are dispensable for viral growth in cell cultures [97]. Moreover, their functions seem to overlap with each other, so deletion or knockout of these genes has been frequent [98]. An ORF7 deletion mutant constituted 87% of the sequences in Australia during the Delta outbreak [99]. However, most other ORF7 deletions are random and sporadic and have never been fixed in any lineage, attesting to the neutral nature of the mutations [99,100]. On the other hand, ORF8 deletions are much more common and repetitive [101]. ORF8 of SARS-CoV-2 encodes an accessory protein to downregulate expression of the major histocompatibility complex I (MHC-I) molecules on infected cells for the purpose of immune evasion [102]. Alpha, Delta, and XBB each inactivated ORF8 in unique ways, accounting for the higher number of fixed mutations in ORF8 [91,98,103]. These lineages caused the most devastating waves of the COVID-19 pandemic. There is evidence that deletion or knockout of ORF8 is positively selected [92,104]. If so, the return of intact ORF8 in the JN.1 lineages after XBB dominance is likely a result of hitchhiking.

Weak selection, hitchhiking, and drifting all contribute to nonadaptive diversification of the nonstructural and accessory genes, which eventually may lead to a reduction in fitness.

The Mistigri Hypothesis on Gene Loss

Adaptive mutations often lead to loss of genetic material, which may be functional in different environments. Multiple in-frame deletions in the N-terminal domain (NTD) of the SARS-CoV-2 spike protein aid in immune escape with no known functional loss [105,106]. Deletion of NTD amino acids 25-27, 31, 69-70, 144, and 212 has been fixed in multiple lineages [53]. Adaptation by genetic streamlining has been proposed as an explanation for the origin of some *Mycoplasma*, *Rickettsia*, and *Chlamydia*, the Ministigri hypothesis [107]. Not only are nonfunctional microbial genes often deleted in natural and laboratory settings, but functional genes may also be lost due to genetic drift [108]. If deletions indeed increase replicative fitness by conserving energy [98], we can see from the repeated, yet hard-to-fix deletions of SARS-CoV-2 accessory genes that the selective force for deletion is relatively weak. Even though deletions may increase fitness in some environments, loss of genetic information decreases adaptive potential. Theoretically, the gene loss of SARS-CoV-2 may reduce its infectivity in non-human host species.

Deoptimization of Codon Usage

Based on expected frequencies of the third nucleotide in four-fold degenerate codons, the fitness effect of synonymous mutations in SARS-CoV-2 was calculated to be neutral and occasionally negative [87,109]. Comparisons between mutation counts in coding and noncoding sequences indicated purifying selection on synonymous mutations [110]. Synonymous mutations affect codon adaptation of the virus to the host’s codon usage bias (CUB). The codon adaptation index (CAI) and the codon pair adaptation index (CPAI) reflect the match of the viral codons and the host’s CUB. When the HIV-1 jumped from chimpanzees to humans, codon usage of the virus progressively adapted to that of the human cell during the first 15 years, followed by deoptimization due to degenerative mutations [84]. Most large sample analyses show that codon adaptation of SARS-CoV-2, especially in the larger genes, decreased until the emergence of Omicron [111-117]. There was a sudden jump in CAI or CPAI in the Omicron variant, followed by a tendency to drop again [110,111,113]. Based on the data of Padhiar and colleagues [117], I found a statistically significant decline in CPAI of the spike gene up to July 2024 [8]. Interestingly, the decline of CAI in Omicron was accompanied by a rise in dN/dS over the same time period [111], suggesting that adaptive amino acid substitutions took priority over codon optimization. Davidson and colleagues analyzed multiple DNA and RNA viruses and found that most viral proteins show declining CUB scores over time [112].

Decline in codon adaptation likely leads to decreased replicative fitness in the host species, but its link to pathogenicity is complicated. SARS-CoV-2 has lower CAI than SARS-CoV-1 and MERS-CoV, which have been thought to contribute to its low pathogenicity [116], but the CAI increase in the Omicron variant was associated with attenuated virulence, presumably due to accompanying nonsynonymous mutations.

Loss of Proofreading Function

Nonstructural protein 14 (NSP14) of SARS-CoV-2 encodes an exonuclease that is responsible for the proofreading function during viral replication [118-120]. Mutations in NSP14 were associated with an accelerated increase in mutation density throughout the viral genome [119]. One missense mutation, NSP14:I42V, has been fixed in the Omicron variant. The mutation has been associated with decreased neuropathogenicity [121].

Preserved under low immune pressure?

Simultaneous emergence of multiple advantageous missense mutations in the Omicron variant, along with an increase in CAI, is puzzling because pleiotropic effects of mutations usually result in functional trade-offs. Even though synonymous mutations may improve codon usage without pleiotropic effects [110], they are vastly outnumbered by missense mutations, which typically sacrifice CUB [88,91,111,122]. The best accepted hypothesis for the origin of Omicron is long-term evolution in a chronically infected immunocompromised host [123]. Most of the mutations in the original B.1.1.529 were not fixed [53]. Such a large number of missense mutations on their way to fixation is not seen in other variants, indicating a unique environment of low selection pressure, which is explainable with the weak antibody response in an immunocompromised host. Low immune pressure may have given the virus time to maintain its original features and even to improve codon usage at non-synonymous mutation sites. It is noteworthy that the “improvement” of codon usage in Omicron was relative to the deoptimized CUB of the Delta variant, but the CPAI of the initial Omicron virus was still comparable to that of the original virus [8,117]. While all other VOCs were identified within one year of the pandemic, Omicron emerged at the end of year two. Since SARS-CoV-2 infections lasting over 500 days have been reported, the origin of Omicron could have been accomplished in a single patient [124,125]. The situation was like the immunotolerance and immunodeficiency in HIV-infected individuals, which gave the virus 15 years of codon optimization before eventual deoptimization [84].

A similar mechanism has been proposed for the sudden appearance of multiple missense mutations in BA.2.86, which had increased CUB scores over the original Omicron [112,125]. However, BA.2.86 did not alter the overall declining trend in CPAI of the spike protein [8,117].

## Conclusions

With its short life cycle, rapid mutation, and strong selective forces, SARS-CoV-2 provided an opportunity to observe evolutionary processes that would take a longer time in cellular organisms. The virus confirmed many evolutionary concepts developed over the past centuries. The rapid emergence of multiple variants demonstrated adaptive radiation followed by selective sweeps and extinction. The nature of adaptation was functional adjustment and specialization, losing fitness in bat cells to gain fitness in human cells, losing fitness in lung cells to gain fitness in nasal cells, and losing replicative fitness to gain epidemiological fitness. Due to pleotropic trade-offs, natural viral evolution in the human population could not improve receptor-binding affinity to the same level as in vitro evolution with isolated molecules. As the receptor-binding affinity approached its natural limit, further mutations yielded diminishing fitness gains, demonstrating negative epistasis, with reversions exemplifying sign epistasis. While the Delta variant outcompeted earlier VOCs and VOIs, its dead-end mutations prevented it from further evolution in the presence of Omicron, which ended up being the only variant evolving toward long-term coexistence with humanity. Positive epistasis was highlighted by the synergistic RBD mutations that reconfigured the receptor-binding interface. Punctuated equilibrium was illustrated by the sudden appearance of receptor-binding and immune escape mutations followed by subsequent antigenic drift. Accumulation of deleterious mutations and a trend of codon deoptimization point to Muller’s ratchet in action. Somewhat surprisingly, low selection pressures in immunocompromised patients may have provided safe havens for adaptive evolution without degradation. 

While some of the history of SARS-CoV-2, such as the origin of the virus itself and of the Omicron variant, may have happened in an obscure corner of the world and remain in the dark forever, we are confident that the uneven access to virus-monitoring technology will not prevent us from witnessing the virus’ future development collectively, because, unlike HIV-1 whose subtypes established separate endemic reservoirs in different countries, the evolution of the airborne respiratory virus has been, and will likely continue to be, global in nature.

## Appendices

Glossary

Adaptive Radiation

An evolutionary process where one species rapidly diversifies into many new species, each adapting to a distinct ecological niche. However, SARS-CoV-2 underwent adaptive radiation early in the pandemic, even in a single ecological niche. This is because of the hostile environment of the human host, forcing the virus to experiment with multiple mutational patterns simultaneously. It was enabled by the error-prone nature of the viral RNA-dependent RNA polymerase.

Diminishing Return Epistasis

The evolutionary phenomenon where the positive effect of a beneficial mutation is smaller when it happens in a fitter organism than in an organism that is less fit. When a species evolves through a series of mutations, the order of the mutations determines the effect of each mutation, with earlier mutations yielding more benefit than later mutations. However, SARS-CoV-2 has demonstrated that the effects of mutations determine the order of their fixation, with higher-impact mutations fixed before lower-impact mutations. This is because of the limited ways to improve a function, forcing the organism to explore lower-impact mutations after the higher-impact mutations have occurred. 

Muller’s Ratchet

Irreversible accumulation of deleterious mutations in asexual organisms as a result of genetic drift. However, SARS-CoV-2 has demonstrated that Muller's ratchet operates even with limited genomic recombination and natural selection. This is because most deleterious mutations are not eliminated by natural selection due to low fitness effects or conflicting pleiotropic effects. 

Path Exclusion

Commitment to one pathway of functional improvement excludes other pathways. Because evolution has no foresight, temporary functional improvement may lead to a pathway with limited future potential. For example, L452R in Delta excluded N501Y, preventing evolution toward optimal receptor binding.

Pleiotropic Effect

A single genetic variation results in multiple phenotypic changes. For example, a spike mutation facilitates immune escape but reduces receptor-binding affinity.

Punctuated Equilibrium

Rapid genetic changes followed by long periods of stasis. The word "equilibrium" initially meant minimal net morphological changes in the fossil record. Here, we focus on genetic and functional changes. While SARS-CoV-2 maintained functional stability between evolutionary bursts, there was no true genetic equilibrium as the virus degenerated irreversibly.

Selective Sweep

A process in which a beneficial mutation quickly goes to fixation due to strong positive selection, resulting in the reduction or elimination of variation. A hard sweep refers to rapid fixation of a single new mutation or a single new combination of mutations, while a soft sweep refers to fixation of a convergent mutation in multiple variants or a pre-existing mutation among the variants. Examples of hard sweeps in SARS-CoV-2 include the D614G mutation, the Delta VOC, the Omicron VOC, and the JN.1 subvariant. Fixation of E484K and N501Y in multiple early variants exemplifies soft sweeps.  As mutations became repetitive and cyclic, later Omicron subvariants experienced more soft sweeps than hard ones.
